# Understanding How Organized Youth Sport May Be Harming Individual Players within the Family Unit: A Literature Review

**DOI:** 10.3390/ijerph111010226

**Published:** 2014-10-01

**Authors:** Corliss N. Bean, Michelle Fortier, Courtney Post, Karam Chima

**Affiliations:** School of Human Kinetics, University of Ottawa, 125 University Private, Ottawa, ON K1N 6N5, Canada; E-Mails: cbean@uottawa.ca (C.N.B.); cpost015@uottawa.ca (C.P.); kchima@rogers.com (K.C.)

**Keywords:** sport, negative, family, youth, parent, sibling, review

## Abstract

Within the United States, close to 45 million youths between the ages of 6 and 18 participate in some form of organized sports. While recent reviews have shown the positive effects of youth sport participation on youth health, there are also several negative factors surrounding the youth sport environment. To date, a comprehensive review of the negative physical and psychological effects of organized sport on youth has not been done and little thus far has documented the effect organized sport has on other players within a family, particularly on parents and siblings. Therefore the purpose of this paper is to conduct a review of studies on the negative effects of organized sport on the youth athlete and their parents and siblings. Articles were found by searching multiple databases (Physical Education Index and Sociology, Psychology databases (Proquest), SPORTDiscus and Health, History, Management databases (EBSCOhost), Science, Social Science, Arts and Humanities on Web of Science (ISI), SCOPUS and Scirus (Elsevier). Results show the darker side of organized sport for actors within the family unit. A model is proposed to explain under which circumstances sport leads to positive versus negative outcomes, ideas for future research are drawn and recommendations are made to optimize the youth sport experience and family health.

## 1. Introduction

Within the United States, 75% of families have at least one child that participates in organized sport [1], while in Canada 76.4% of youth participate in extra-curricular sports [2]. Further, within the United States close to 45 million youth between the ages of 6 and 18 participate in some form of organized sport [3]. With an increased emphasis on sport in today’s society, and as sport is a predominant avenue in which youth spend their leisure time, it is important to understand the impact this participation has on the youth themselves and their family members. Research has consistently identified the family as a critical influence on youth throughout the socialization process of sport participation [4,5,6]. Moreover, a plethora of previous research has investigated the influence of family on youth sport involvement [7,8,9,10,11]. Most recently, Fraser-Thomas, Strachan, and Jeffery-Tosoni [12] wrote a chapter aiming to understand the influence of family on children’s involvement in sport. It was found that parents have a significant influence on youths’ participation, performance and enjoyment in sport and identified different types and levels of parental support. Moreover, they revealed that siblings can have both positive and negative effects on the youth athlete, such as being supportive and acting as a role model, but also conversely experiencing feelings of isolation, bitterness and jealously. While this chapter provided much benefit in understanding the impact of the family on the athlete, little to date has documented the reverse relationship that is the effect of organized sport on individual players within a family, particularly parents and siblings. Moreover, much literature is available on the positive aspects of sport for youth, including physical and psychological effects [13,14,15,16]; however, to our knowledge, a review of the darker side of the youth sport experience has not been conducted, particularly surrounding the negative impact organized sport has on the actors within a family unit (e.g., the youth athlete, their parents, their siblings).

Despite a lack of comprehensive scientific investigation into the bleaker side of the organized sport experience, this issue has been well-documented in the media in recent years. For example, the New York Times [17] ran an online debate entitled “Can Playing Ball be Bad for Kids?” addressing many of the issues in the sporting environment that appear to be doing more harm than good. Coaches, parents and other stakeholders discussed topics such as injuries, burnout, over-scheduling, and pressure to succeed. These issues can negatively influence not only the physical and psychological health of our society’s youth, but also the well-being of their families and should thus be of high concern in the domain of public health.

With regards to the negative impact of organized sport on youth, Jayanthi *et al.* [18] recently provided evidenced-based recommendations related to early specialization in youth athletes. The authors primarily reviewed the detrimental physiological and some psychological effects of early sport participation, such as dropout, indicating that while some degree of sports specialization is important in order to develop skills for elite-level sport, intense training in a single sport should be postponed until youth have reached late adolescence in order to optimize success while also minimizing physical injury [18]. Conversely, this paper failed to explore many of the psychological effects of sport participation, such as substance abuse and burnout. Additionally, the purpose of their review was mainly to provide recommendations for practical application. Finally, they did not discuss the effects organized sport participation may exert on family members other than the youth athlete. Therefore, the purpose this paper was to conduct a comprehensive review of literature on the negative effects of organized sport on the individuals within the family unit: the youth athlete, their parents and their siblings. More specifically, this review aimed to understand; (1) What are the negative physical and psychological effects of organized sport on the youth athlete?; (2) What are the negative financial, practical, and emotional/psychological effects that parents experience with their involvement in organized youth sport?; (3) What are the negative psychosocial effects of organized sport on sibling(s) of youth athletes? Although significant strides have been made in recent years to better understand the darker side of the organized sporting experiences, there is still much ground to cover. This review is a novel undertaking as the vast majority of the literature has looked at the positive effects of sport on youth athletes [16,19] and very little has been written on the effects of organized sport on other family members.

This review is divided into three main sections with the goal of extensively investigating the negative physical and psychological influences of organized sport on the three main actors within a family: youth athletes, parents, and siblings. For this paper, “organized sport” (hereafter referred to as “sport”) is classified as involving formally structured physical activities governed by sets of rules and takes place under the presence or instruction of direct adult supervision (e.g., coach). Organized sport is voluntary, yet often typically competitive that is structured into leagues and/or competitions and usually takes place as either school sport, community sport, or club sport [20,21,22,23]. Youth, within this review, are considered to be individuals that fall between childhood and adulthood ranging from 6 to 18 years of age. Finally, the family unit consists of one or more youth within the aforementioned age range who is/are involved in sport, his/her parent(s), and his/her siblings.

## 2. Methods

### Literature Search Procedures

The main goal of the search procedures was to locate and review all empirical studies, meta-analyses, literature reviews, book chapters, or doctoral dissertations on the physical and psychological effects of youth sport on the individual actors of the family unit. For the sake of paper length, the social impact of organized youth sport, such as violence and sexism, was not reviewed within this paper. These retrieved sources could use any data type (quantitative or qualitative) and any design (e.g., surveys, randomized group designs, interviews) to review the literature surrounding the negative effects of organized sport on the individual actors within the family unit.

The following procedures were used to locate sources: (a) computer searches of four databases (Physical Education Index and Sociology, Psychology databases (Proquest), SPORTDiscus and Health, History, Management databases (EBSCOhost), Science, Social Science, Arts and Humanities on Web of Science (ISI), SCOPUS and Scirus (Elsevier)) using variants of the following search terms in terms of vocabulary and combinations: youth (child, adolescent), organized sport (sport participation), negative, family, sibling (brother, sister), parent (mother, father); (b) inspection of the reference lists of relevant articles; and (c) manual searches (e.g., using Google Scholar) in attempt to find any additional relevant articles that did not arise during previous searches. Initially, searches were not restricted by publication date; however, upon completing the search, a cut-off date of 1999 was established in order to ensure analysis for this review was manageable. In addition to a publication date of no earlier than 1999, the inclusion criteria for the review was that literature retrieved had to be published in English and that the title, abstract, or keywords had to contain at least two or more of the search terms. Lastly, the study sample had to fall within the age bracket of 6 to 18 years of age, which corresponds to the Development Model of Sport Participation which is discussed in the following section [24].

Once the research team had completed the literature search that fit within the inclusion criteria, 185 articles were retrieved and entered into an Endnote database. The full texts of these 185 articles were reviewed by three authors; each reviewing articles related to one component of the family unit (youth athlete, parents, siblings). Upon completion of initial individual analysis, the four authors met and discussed any discrepancies or gaps within the reviewed literature until an agreement was reached. This process helped to ensure that a comprehensive review was conducted and that the results were accurately represented. Literature that covered more than one actor in the family unit was integrated into both sections of the review when relevant and not redundant. Media sources (e.g., magazine, newspaper articles) were used within the discussion to support academic literature, but were not included in the total number of academic papers comprehensively reviewed for this review. All literature searches took place between the months of January and April 2014. Due to the variety of measures and study designs employed across different studies, it was not possible to perform a meta-analysis or meta-ethnography; therefore, a narrative synthesis [25] was employed. Any additional information surrounding the search procedure can be attained from the corresponding author.

## 3. Results

The results are divided into three sections based on the family unit: (1) youth athlete; (2) parents; and (3) siblings. In the first section of this review the negative effects of sport participation on the youth athlete are considered, including physical and psychological effects. The second section focuses on the influence youth sport participation has on the parents within the family, such as time and financial constraints, as well as parents taking on multiple roles (e.g., coach). Thirdly, the negative effects of the sport environment on siblings of youth athletes are examined, particularly focusing on the psychosocial effects. Lastly, the authors discuss limitations of this study, as well as current gaps in the literature, leading to calls for future research and practical applications based on the literature reviewed.

### 3.1. The Effects of Organized Youth Sport on the Youth Athlete

In recent years, the balance between competitive sport and youth well-being has received much scrutiny in both the media and in peer-reviewed research. For example, the New York Times recently ran an online debate on children’s sport-life balance [17]. In this forum, some experts stated that competitive sport has become too serious, competitive and adult-driven, while others insisted that sport is the best way to teach youth important values such as discipline and teamwork [17]. Additionally, within peer-reviewed research, concerns have been raised over the physiological [26] and psychological [27,28] stresses that are often associated with youth sport. The ample, and somewhat troubling findings presented on these topics in the media and within academia indicate that the state of youth sport has become an issue of great concern for all those involved and thus a review on this topic is timely and of great public health importance.

In the following sections, research detailing the impact organized sport exerts on the physical and psychological health of youth is reviewed. First, an outline of Côté *et al.*’s [24] Developmental Model of Sport Participation (DMSP) is provided, which describes the various pathways young sport participants follow between the ages of 6 and 18 [29,30,31]. This model is used throughout the paper when discussing the “early sampling” *vs.* “early specialization” pathways.

#### Developmental Pathway of Sport Participation and the Sampling *vs.* Specialization Debate

The developmental pathway followed by a youth athlete is an extremely important component in the complex puzzle of what defines the youth sport experience. The different pathways have been described in Côté and colleagues’ [24] DMSP. According to the DMSP, athletes can follow one of three developmental pathways between the ages of 6 and 18 years: (1) consistent recreational participation, where the athlete does not specialize in any one sport; (2) early sampling of a variety of sports before later specializing in adolescence; and (3) early specialization, where the athlete engages in high amounts of deliberate practice upon entry into the sporting world [24]. In the past two decades, the literature has been marked by the “specializing *vs.* sampling” debate, as both of these pathways have been shown to lead to the end goal of elite performance [24,29,32]. Increasingly, however, youth sport programs have been moving towards encouraging athletes to devote themselves to a given sport at a very young age (e.g., early specialization) in hopes of gaining an advantage over the competition and making it to elite levels. This practice has raised developmental concerns about negative outcomes that may result from such intense practice early in a child’s life [18]. Experts have recently concluded that intense training in a single sport to the exclusion of all others should be delayed until late adolescence, for physiological and psychological health reasons alike [18,33,34,35].

### 3.2. Effects on Youth Physical Health

As earlier mentioned, Jayanthi *et al.* [18] discussed negative effects of early sport specialization including higher rates of injury, increased psychological stress, and dropout at a young age. The following sections provide a review of studies on the negative physical and psychological effects of organized sport practice on youth.

#### 3.2.1. Injuries

One of the most commonly studied physical outcomes of early specialization and intense training in sport is athletic injury [26,36,37]. It has been argued that sports are the leading cause of injury in adolescents in Canada [38,39] and similar rates are evident within the United States [40,41,42]. It has been estimated that over two million children and adolescents are injured each year due to sport involvement [40,43]. Further, there are two main categories of injuries that occur during athletic competitions: overuse injuries and impact injuries. In the interest of length of this paper, the only impact injury discussed is the most commonly experienced in sport: concussion; however, see McIntosh [44] or Finch, Ullah, and McIntosh [45] for review of other impact injuries in sport.

##### 3.2.1.1. Overuse Injuries

“High-risk” overuse injuries are defined as those that can result in significant loss of time from sport and/or threaten future sport participation. These include certain stress fractures, physical stress injuries, osteochondritis dissecans, some apophyseal injuries, and effort thrombosis [3]. In a review investigating the physiological concerns associated with organized sport participation in young people, Kaleth and Mikesky [37] reported that overuse injuries were the most common injury seen in youth athletes. These result from specific, repetitive movement patterns that damage a tissue structure [3,37,46] and are most commonly associated with intense single-sport training [34]. The prevalence of overuse injury varies by the specific sport, ranging from 37% of athletes (in skiing and handball) to 68% of athletes (in running) [3].

Additionally, associations have been made between increased participation in organized sport, the tendency towards early specialization, and the increased prevalence of overuse injuries [34]. These links were demonstrated in a study conducted by the American Academy of Pediatrics [46], with a sample of more than 1200 child and adolescent athletes where it was found that athletes ages 8 to 18 who spent twice as many hours per week in organized sports than in free play, particularly when in a single organized sport, were more likely to be injured. Jayanthi *et al.* [18] also found that injured athletes reported a higher average number of hours per week playing organized sportand a higher average number of hours per week in total sports activity including gym, free play and organized sport. Injured athletes also scored significantly higher in terms of sports specialization than uninjured athletes, even after adjusting for hours per week in total sports activity and age [18]. Several other studies have reported similar findings [26,47], which have led researchers to conclude that early specialization and high volumes of organized sport participation increase youth athletes’ risk of injury.

Further, in a study conducted by Luke and colleagues [48] that investigated the association between over-scheduling and sport-related overuse injuries in youth athletes, the authors found that over-use injuries were encountered most over fatigue-related injuries, indicating youth athletes did not have enough rest prior to sport participation, and that parent and athlete perceptions of excessive training without adequate rest was related to both overuse (*p* = 0.016) and fatigue-related injuries (*p* = 0.010). It was also found that sleeping six hours or less the night before an injury was related to fatigue-related injuries (*p* = 0.028). Finally, the authors developed a working definition of “overscheduling injury” which they defined as “an injury related to excessive planned physical activity without adequate time for rest and recovery, including between training sessions/competitions and consecutive days” [48].

Furthermore, it is important to note that not all sports carry the same risk of injury. For example, in their study of adolescent athletes from sports schools, Theisen *et al.* [49] found that injury incidence was significantly higher in team compared with individual sports, when considering both traumatic and overuse injuries. However, there is no definitive data on youth athletes who withdraw from sport because of injury [50,51]. Despite this, there is a general consensus that regardless of the sport, early specialization increases the injury risk of the young athlete [3,18,26,52]. In order to prevent such injuries from occurring it is recommended that there be a link between an athlete’s level of growth and development and the demands of the sport in question, and that young athletes play multiple sports in childhood [3,18,53].

##### 3.2.1.2. Concussions

In addition to overuse injuries commonly associated with intense training in one sport, concussions have become a growing concern in both academia and the media. Indeed, concussion injuries have received ample media attention in the past five years, particularly in the sports of hockey and football (e.g., [54,55,56]). Some reporters have even gone as far as labeling contact in minor hockey as a form of child abuse [57]. The frequency of these injuries in sport participants, which has been estimated at 3.8 million concussions per year in the United States alone [58], has given reason to describe the situation as an “epidemic” [59]. Additionally, 25%–30% of all mild traumatic brain injuries seen in the emergency department in 5–14 year olds are sports-related or bicycle-related injuries, and concussion accounts for almost 15% of sport-related injuries in high school athletes [58]. In their study that examined concussion rates, Marar *et al.* [60] found that young athletes who are most at-risk for these injuries are those who participate in football (6.4 concussions/10,000 athlete exposure), boys’ hockey (5.4) and boys’ lacrosse (4.0).

Moreover, there is mounting evidence that youth are at greater risk for concussions than the general population [61,62,63] as youth tend to take longer than adults to physically and often psychologically recover following a concussion [63]. Suffering from a concussion has been shown to result in decreased cognitive functioning, such as memory loss. While the long-term impacts of multiple concussions and repetitive head injuries are not fully understood, initial evidence has indicated that multiple concussions sustained in youth can lead to long-term neurodegenerative diseases, such as chronic traumatic encephalopathy and Alzheimer’s disease [64]. Moreover, these effects can impact one’s quality of life and the ability to carry out one’s daily activities [64].

In response to this knowledge, governing bodies of youth sport organizations have directed their focus towards emphasizing the return-to-play guidelines [47,65,66] and United States laws surrounding youth sports traumatic brain injury. Return-to-play guidelines are a set of recommendations detailing the steps an individual must follow before returning to their sport after sustaining a mild traumatic brain injury. This need has prompted governing bodies of sport organizations to take steps towards educating athletes, parents, and coaches about the dangers of concussion injuries (e.g., Hockey Canada Concussion Application, Sports Trauma and Overuse Prevention (STOP) Campaign) [67,68]. Although this represents an important, concrete step towards alleviating this serious problem, awareness is not prevention [69]. Harvey [69] found that 44 states within the United States have passed youth sports traumatic brain injury laws between 2009 and 2012; however, no state’s laws focus on primary prevention. Instead, such laws can be applied only after a concussion has been sustained, such as focusing on increasing coaches’ and parents’ ability to identify and respond to brain injuries in youth athletes and reducing the immediate risk of multiple brain injuries. More research is needed in order to test whether or not these programs really do help to prevent concussions from occurring in youth sport [69,70]. Researchers and health professionals alike are suggesting that more attention should be placed on rule alterations and legislation in order to make contact sports safer and more developmentally appropriate for youth participants [65,71,72].

A critical rule change suggested by sport experts was delaying the age at which contact is allowed in sports such as ice hockey [65,73]. In line with this, the American Academy of Pediatrics has recommended that body checking in ice hockey be limited to players over the age of 15 [72]. Despite this recommendation, most ice hockey associations across North America allow body checking as early as 13 years old [74]. This particular issue reinforces how the culture of sport in today’s society is engulfed in the idea of performance and professionalization [65], which ultimately has a negative impact on the physical health and well-being of young athletes.

##### 3.2.1.3. Nutrition and Weight Control

Consuming a healthy and balanced diet is of great importance for young people [75], especially young athletes [76]. However, recent research has emerged indicating that the youth sport experience may exert a negative impact on the food choices made by athletes and families [77,78]. In their mixed methods study that included in-depth interviews with 47 different families, Chircop *et al.* [77] found that increased emphasis on participation in sports was associated with more fast food consumption. It was shown that due to time constraints associated with the organized sport experience, families reported eating fewer meals at home and placed more emphasis on physical activity participation than on healthy eating. Similar to these results, Tomlin *et al.* [79] conducted a cross-sectional descriptive study involving 1,421 school-aged children and found that children who were involved in organized sport consumed more fat and calories than children not involved in sport. However, it was concluded that children involved in these sports were more physically active, consumed a healthier diet and had lower BMIs than youth who were not athletes, despite consuming more calories.

Similarly, in Nelson and colleagues [80] systematic review, they found that in studies which compared eating habits of sport participants *vs.* non-sport participants, youth involved in sport were more likely to consume fruits, vegetables, and milk, and also more likely to eat fast food and drink sugar-sweetened beverages and consume more calories overall. Given the importance of healthy eating throughout the developmental years of childhood and adolescence [76,81], it is clear that more research is warranted to better understand the links between organized sport, food consumption and optimal health.

Closely related to the topic of nutrition is the issue of weight control. In a recent position statement on safe weight loss and maintenance practices in sport and exercise, Turocy *et al.* [82] reported that athletes often attempt to lose weight by not eating, limiting caloric or specific nutrients from their diet, engaging in pathogenic weight control behaviours, and restricting fluids. These athletes often attribute their actions to pressures placed on them by the sport, their coaches, peers, or parents [82]. These authors also suggested that due to increases in organized sport participation and intensity of competition at an early age, there is a renewed interest in controlling all factors related to performance and health. Thus diet, exercise, body composition, and weight management now play larger roles than ever before in an active person’s life and performance [82,83].

In a review conducted by Ricciardelli and McCabe [84], it was found that factors associated with eating disorders in adolescent boys are similar to those found with girls, such as body mass index, negative affect, self-esteem, perfectionism, drug use, perceived pressure to lose weight from parents and peers, and participation in sports that focus on leanness. Sports that are encouraged for girls prior to the onset of puberty, such as gymnastics or figure skating, often involve high risks of eating disorders and amenorrhea [85]. The prevalence of female youth athletes who have eating disorders ranges between 20%–26% [86,87,88,89,90]. When compared to control groups, it has been found that female youth athletes experience a higher frequency of menstrual dysfunction, ranging from 0%–40% [91,92,93] and reaching 65%, depending on the type of sport and the intensity of training [94]. The issue of weight control is of primary concern in adolescent sports that involve a weight classification system or place strong emphasis on weight, such as youth football, wrestling, rowing, and boxing (see [95] for review). For example, 85% of female youth who participated in weight-class sports, reported attempting to reduce their weight compared with endurance and ballgame athletes and controls (29%–58%) [96]. While weight and body composition standards exist for these activities [97], there continues to be confusion surrounding best practices balancing performance and health in these type of sports [98]. This confusion is mirrored in other weight-sensitive sports (e.g., dance, distance running, figure skating and cycling ) [82,98].

The lack of regulations and general knowledge regarding healthy weight control methods may be one of the reasons why some studies have found that adolescents involved in high-level sport are more at-risk of developing an eating disorder than adolescents who are not involved in competitive athletic activities [84,99,100]. In their study comparing eating disorder prevalence in athletes (n = 1620) to a control group of non-athletes (n = 1696), Sundgot-Borgen and Torstveit [101] found that more athletes (13.5%) than controls (4.6%; *p* < 0.001) had subclinical or clinical eating disorders. Further, athletes were more likely to develop an eating disorder if they participated in sports in which (a) weight classifications apply (e.g., wrestlers, rowers), (b) weight or small body size is important for success (e.g., distance runners, cyclists), or (c) subjective evaluation and aesthetic ideals co-exist (e.g., figure skaters, gymnasts, divers) [102]. The physical consequences of eating disorders in athletes can have long lasting negative impacts, including decreased athletic performance and overall health and well-being [100]. These illnesses also have high-mortality rates [103].

This section has reviewed the common physical health concerns associated with participation in youth sport. The following section focuses on the research that has examined the ways in which sport may be negatively impacting the psychological health of young people.

### 3.3. Effects on Youth Mental Health

More often than not, when the term “mental health” appears in academic literature it is discussed in terms of mental illnesses such as anxiety or depression (e.g., [104,105]). However, in the past 15 years there has been a push from scholars who argue that describing mental health this way only illustrates a portion of the story [106,107,108,109]. Keyes [106] argued that mental health is a complete state, not merely the absence of disease but also the presence of sufficiently high levels of well-being referred to as “flourishing”. Keyes proposed a two continua model of mental health/mental illness and has developed and validated a measure known as the Mental Health Continuum–Short Form (MHC-SF), which he [106,110,111,112], and other researchers [107,113,114,115], have used to accurately classify individuals according to their current state of mental health. Just as an individual needs to demonstrate symptoms of malfunctioning to be diagnosed as depressed, an individual must also demonstrate symptoms of high functioning in order to be classified as flourishing, the highest level of mental health [106]. Specifically, in order to be classified as flourishing, an individual must exhibit high levels of emotional, psychological and social well-being [106].

Mental health in the general youth population has been of growing concern in the past two decades, with suicide and depression rates on the rise [116,117]. In his study of 12–18 year olds, Keyes [110] found that those who were classified as flourishing, as opposed to moderately mentally healthy or languishing, had the lowest incidence of conduct problems such as skipping school, being arrested, smoking cigarettes, smoking marijuana and drinking alcohol. Additionally, flourishing youth also reported the fewest depressive symptoms and the highest levels of global self-concept, self-determination, closeness to others and school integration [106].

#### 3.3.1. Well-Being

To the authors’ knowledge, there are no published studies applying the positive psychology perspective on mental health to youth sport. However, there has been considerable research linking sport involvement to well-being [15,118]. For example, reviews have concluded that the sport environment, particularly team sports, can help to ease symptoms of psychological ill-being, while also being a positive source for the development of youth well-being, such as increased self-esteem and social interaction [15,118]. Moreover, in their quantitative study involving 1633 children in grades four to six, Holder *et al.* [119] found that active leisure, such as sport, was related to well-being, while passive leisure, such as watching TV, were not. Further, there is a large body of research surrounding positive youth development which has consistently shown that, if structured appropriately, organized sport can promote optimal development in youth [120,121,122,123]. Despite these findings, recent evidence has brought to the forefront the issue of the volume of sport practice required to optimize well-being. In a recent study examining the relationship between the quantities of sport practice engaged in per week and well-being, it was found that after 14 hours per week of sport participation, well-being began to decline in adolescents [124]. These findings parallel the work done by Brown *et al.* [105] that examined the influence of physical activity on quality of life in adult populations. Based on these results, as well as others, it appears that while some participation in sport is good for well-being [15,105], too much can have a detrimental effect.

Beyond simply considering the volume of sport practice, there are several other factors involved in determining whether the sport experience can help or harm the well-being of a youth athlete. The following sub-sections further delve into the other aspects of this pertinent issue, such as the relationship between sport and alcohol use and substance abuse, burnout and dropout, and motivation.

#### 3.3.2. Alcohol Use and Substance Abuse

While the use of drugs and alcohol do have negative physiological effects on youth athletes, these substances have also been a predominant issue within the literature surrounding negative psychological effects. The role that sport participation plays in the use of alcohol has been an area that has received focus within academic literature. In their recent systematic review of 17 longitudinal studies that examined this relationship, Kwan *et al.* [125] found that sport participation is associated with alcohol use in the adolescent population, as 82% of the studies reviewed showed a significant positive relationship. The researchers also found a significant interaction between socio-economic status and sport participation. Specifically, the findings suggested that sport participation was associated with increased overall alcohol use during high school, but that the relationship appeared stronger for youth living in higher (*vs*. lower) socio-economic neighborhoods. Further, in a study conducted with Icelandic secondary students, Halldorsson *et al.* [126] compared alcohol use throughout adolescence in three different groups: adolescents involved in formally organized sport clubs (referred to as ‘organized sport’ in North America), those involved in informally organized sport activities, and those not involved in sport. Results revealed that youth who participated in formally organized sport clubs were less likely to use alcohol than those who did not participate in these clubs. However, it was found that adolescents that participate in only informal sports are more likely to use alcohol than those that do not. These findings suggest that participating in sport might not necessarily lead to alcohol use in teens, but certain sporting environments may lend themselves to more frequent use of alcohol. In line with this, a longitudinal study conducted with Norwegian high school students indicated that sports participation in adolescence, particularly with team sports over individual sports, increased the use of alcohol during late adolescent into early adulthood [127]. Further, this study showed that youth in technical sports such as ice hockey, soccer, or football have greater increases in alcohol use over time compared to those who participate in endurance sports such as running, speed-skating, or swimming, or strength and power sports such as boxing, wrestling, or gymnastics [127]. Martens *et al.* [128] found that out of a sample of 298 intercollegiate athletes, individuals within swimming and diving sports reported the highest levels of alcohol consumption. Lastly, in a systematic review that examined 6890 studies across the United States, the results indicated higher rates of alcohol use and violence in athlete populations when compared against non-athlete populations [129]. It was concluded that masculinity, violent social identity and anti-social norms were linked to traditional masculine sports (e.g., ice hockey, football) and may have an overall impact on the association between sport and violence in athlete populations. [127,129]. Caution must be taken when interpreting these findings as it is possible that athletes that are attracted to rough team sports might also be attracted to alcohol and other substances; therefore, it might not be their sport participation but their personality that leads to alcohol use.

In addition to alcohol use, research has indicated that youth sport participation is related to the use of other substances, such as marijuana, tobacco, and steroids. However, many studies have optimistic findings indicating that participation in sports at the high school and college levels is linked with lower rates of illegal drug use, such as marijuana and cigarette use [125,126,130,131,132]. This has been found to hold true during high school sport participation and longitudinally, in late adolescence and early adulthood post-high school [126]. It is important to note that this relationship differs by type of drug and type of sport [132]. Moreover, it has been found by Lisha *et al.* [131] that male athletes had significantly higher rates of marijuana use and had decreased rates of alcohol use over time, while female athletes had significantly greater rates of alcohol use over time. Mixed results have been found for youth sport participation surrounding the use of tobacco [126,133,134] and steroids [130,135].

#### 3.3.3. Burnout and Dropout

Burnout is a phenomenon that is considered to be part of a series of negative conditions that include over-reaching and overtraining. It has been comprised of three dimensions that include emotional exhaustion, depersonalization, and reduced performance accomplishment [136] and can be described as a prolonged response to chronic emotional and interpersonal stressors [137,138]. It has been found to occur due to chronic stress that can lead to a cessation of participation in a sport or activity (e.g., dropout) that one had previously found to be enjoyable [3]. Consequently, gaining a better understanding of why burnout occurs and what can be done to prevent it has been viewed as an important issue in the sport psychology literature [139,140]. Typically, athlete burnout is characterized by a loss of motivation shown as reduced intrinsic motivation or amotivation, a lack of enjoyment, possession of poor or ineffective coping skills, high perceived stress and anxiety, and mood disturbance associated with responses to training and non-training stress and insufficient recovery [19,140]. It has been estimated that between 1%–9% of elite adolescent athletes suffer from burnout [141]. Furthermore, there is some evidence to suggest that athletes who specialize early in their sport are more likely to experience burnout than those who follow an early sampling pathway [142]. Results from this study suggested that three developmental assets (positive identity, empowerment, and support) were key elements of focus for youth sport programs in order to decrease burnout symptoms and enhance overall enjoyment. Indeed, according to Gould [143] characteristics related to burnout include extremely high training volumes and time requirements, demanding performance expectations (self- or other-imposed), and continual competition. Personal factors such as perfectionism and a singular focus on athletic involvement have also been identified as precursors to burnout [143,144,145,146]. In relation to youth sport, burnout has been identified as one of the primary risk factors associated with early specialization [18,147]. Because of this established relationship, some researchers (e.g., [34]), have suggested that a child should be at least six years old before playing on a sports team.

The phenomenon of dropout is closely related to that of burnout. Although not all cases of dropout in youth sport are caused by burnout, the former is certainly related to the latter [148]. Additionally, studies have shown that athletes who specialize early in their sport are more likely to dropout [149,150,151,152]. For example, in their study examining the relationship between youth sport specialization and physical activity in young adulthood, Russell and Limle [153] found that sport participation patterns in young adults differ according to the sport specialization status showing that young adults who specialized during their youth sport career were less likely to be involved in sport in adulthood. Further, in a study of youth ice hockey players, Wall and Côté [150] found that while both active and dropout players invested similar amounts of time in the sport, dropout players began off-ice training at a younger age and invested more hours per year in off-ice training, highlighting that engaging in such activities at a younger age may have negative implications for long-term ice hockey participation. Such results are consistent with previous research and seem to indicate that early involvement in practice activities that are not enjoyable may ultimately hinder the intrinsic motivation to continue in sport [150]. Lastly, in a review that investigated correlates of youth sport attrition, the authors found that age, autonomy, perceived competence, relatedness, and task climate were considered to be high quality correlates, where, correlates that were considered of low quality included attributing success to external sources, conflict between sport and non-sport activities, intention to participate in sport, and positive expectancies of future in sport [154]. It should be noted that correlates related to youth sport attrition tend to be social in nature. The following section discusses the importance of motivation for youth athletes.

#### 3.3.4. Motivation and Motivational Climate: Some Missing Links

Some researchers have turned to Self-Determination Theory (SDT) [155,156] as a means of explaining both the positive and negative sides of the sporting experience [19,33,157]. It has been well-established in the literature that when an athlete’s needs for competence, autonomy and relatedness are met, self-determination is fostered and positive outcomes such as high vitality, positive affect and increased self-esteem result [157,158]. Conversely, when these basic needs are thwarted, maladaptive outcomes such as disordered eating, burnout, depression, negative affect, and perturbed physiological functioning occur [159]. Because the decision to specialize in a given athletic pursuit at a young age is rarely made by the athlete himself or herself, it has been postulated that athletes who have followed the early specialization developmental pathway may exhibit motivation that is less self-determined than athletes who have specialized later or who have not specialized at all [33]. This could then lead to a host of negative outcomes such as poor mental health, burnout and dropout.

Merkel [34], like many others (e.g., [160,161,162,163,164]), has found that the most common reason youth play sports is to “have fun” (for intrinsic motivation). When sport is no longer fun, dropout is often the result [34]. Indeed in two large longitudinal studies, Pelletier *et al.* [165] and Sarrazin *et al.* [166] both found that self-determined sport motivation (including intrinsic motivation) leads to more sport persistence over time. A similar but more recent study [167] revealed that the most significant predictor of burnout in adolescent swimmers was lack of enjoyment (which is akin to low intrinsic motivation). Further research is needed in this area in order to determine if specialization (or level of sport) influences self-determination and subsequent outcomes. An early study conducted by the second author of this paper [168] did show that competitive athletes were less self-determined than recreational ones, giving weight to this link.

Another factor to consider in this relationship is the role of the motivational climate. SDT is central to understanding the motivational climate found in a given sporting environment [169]. This theory specifies that contexts which support basic psychological needs produce higher quality motivation and positive outcomes than environments which neglect these needs. Recent research has found that perceptions of autonomy support predicts need satisfaction and in return positive outcomes such as vitality and positive affect whereas control is associated with need thwarting and negative consequences [159] A second theory that has been used to explain motivational climate in athletic settings is Nicholls’ [170] Achievement Goal Theory (AGT). According to Nicholls [170,171], motivational climates can be perceived as either task-oriented or ego-oriented. A task-oriented climate is characterized by individuals’ perceiving that their best efforts are encouraged and recognized, cooperation is fostered, and everyone plays an important role. Alternatively, in ego-oriented climates, individuals perceive that only participants with superior ability are recognized and valued and that negative attention is drawn to those who make mistakes [170,172]. It has been confirmed that within the context of youth sport, perceptions of a task-oriented climate are more conducive to overall positive experiences and perceptions of an ego-oriented climate are more likely to promote negative outcomes [173]. Both SDT and AGT have been widely applied to examine the motivational climate of a variety of sport and physical activity settings (see [169] for a review).

Coaches and parents play important roles in creating the motivational climate of the sporting environment [169]. When an athlete perceives that their coach is fostering a task-oriented climate, this positively predicts the satisfaction of the three psychological needs [174]. MacDonald *et al.* [175] found in a sample of 510 athletes between 9 and 19 years old that positive experiences in sport were most strongly predicted by affiliation with peers, self-referenced competency, effort expenditure, and a task climate. Negative experiences were most strongly predicted by an ego-oriented climate. This has been re-enforced within the literature indicating that task-oriented climates are associated with higher enjoyment and lower anxiety in sport [176,177]. Furthermore, the quality of child-parent relationships is an important predictor of young athletes’ stress, level of enjoyment, and self-determined motivation [9,178]. Parent behaviour construed as negative, coercive, or as communicating excessive evaluative concerns (*i.e.*, an ego-oriented or controlling style), has been shown to contribute to a more threatening sport performance environment [179,180,181,182]. Such behaviours are frequently part of the ‘win-at-all costs’ mentality that increases stress and reduces enjoyment in youth sport [183]. Research has also found that parental pressure which is related to an ego-oriented climate or controlling style has been linked to higher performance anxiety and negative affect in young athletes [184]. The research surrounding this topic has been consistent in establishing the important influence coaches and parents exert on young athletes [169]. It is quite likely that the motivational climate created by coaches and parents moderates the effect that sports has on various outcomes such as athlete well-being. Future research needs to test this and coaches and parents would be well-advised to take these findings into consideration in their interactions with young athletes, this is elaborated upon in the discussion, whereas in the next section, we review literature associated with how a parent’s involvement in their child’s sporting experience can negatively impact them.

### 3.4. The Effects of Organized Youth Sport on Parents

Current literature has indicated that parental commitment to youth sport is a key factor in youth sport participation [35,185,186,187]. By encouraging continued participation, teaching values and norms associated with sport participation and providing opportunities to observe and participate in sport, parents influence their children and facilitate their socialization within the sport domain in a variety of ways [188,189]. That said, there is little research pertaining to the impact of the youth sport experience on parents themselves. However, what is known is that the youth sport environment also influences parents and their way of life [4,190]. Recent studies have taken on a more parent-centric perspective that examined the effects of youth sport on parents [35,191,192,193]. The following sections discuss both the financial investments and time commitments parents often endure for their children involved in sport. Further, research is also reviewed surrounding the effects of youth sport on parental mental health and stress, parents playing dual roles, the challenges for parents associated with multiple athlete families, the burden often faced by mothers in multiple roles, and maintaining a healthy lifestyle and couplehood.

#### 3.4.1. Parents’ Financial Investment Related to the Youth Sport Experience

Many parents see their children’s participation in sport as a vital part of their child’s socialization process [34,194]. Parents often provide instrumental support to meet the needs that arise with their child’s sport participation, including financial costs. These financial costs are often associated with registration and enrolment, equipment and uniforms, travel costs, and private camps [35,195,196]. Early research has indicated that there are substantial financial demands for parents with youth involved in sports that affects their personal, social, and family life choices [197,198].

Once involved in youth sport, it has been found that parents often feel obligated to continue to financially invest in their child’s sport, concerned that if they stop, they deprive their child of their dreams [4,194]. It has been documented that, within Canada, parents place a priority on spending for their children’s sports instead of saving for their college or university education, and have even gone as far as using portions of retirement savings to fund their children’s extra-curricular activities [199]. While these statistics have been highlighted within Canada, it has been argued that there are thousands of families within the United States that are in similar financial situations [200,201,202].

Furthermore, youth sport programs have become increasingly expensive over the years [203,204,205]. For example, for youth recreational ice hockey players, hockey-related expenses are an average $1500 per year. Parents of competitive minor hockey players can spend between $8000–$15,000, as equipment alone can cost upwards of $1200 [206]. These costs are not only associated with ice hockey, but other sports as well [199,200,202,207] and do not account for the registration and league fees that are also required. Research has indicated that parents who have monetary resources are in a better position to buy athletic equipment and pay for special camps and teams than parents who are struggling financially [208]. This is especially important when considering that the demands on the family unit are likely to increase as children age and their level of competition or achievement rises [209]. Such demands often lead to greater financial responsibility for parents, more work hours required and less time to spend with family, which can increase parental stress [4,194,210].

#### 3.4.2. Parents’ Time Commitment Related to the Youth Sport Experience

Participation in athletics requires a significant time investment by the family [211]. This time investment can include travelling to and from events, watching the events, coaching, volunteering, and fundraising [35,187,195,196]. For example, transportation to and from training and competitions, being present during the training or competition, and adapting family routines have all be documented as causing stress to one or both parents within a family [203,210,211]. Some parents have reported devoting up to 20 hours per week to their child’s sport programs [212]. Such an investment in time can foster a sport-work role conflict and a sport-family role conflict for parents [213]. For example, sports have been found to affect typical family life patterns [4]. For example, the number of practices one child’s sport can dictate the schedule of the entire household. Holidays must be scheduled around competitive schedules and training, cancelled, or negated [209,210]. Due to the often rigid scheduling, parents’ employment patterns can also be affected. A study conducted by Kay [209] found that a child’s sport participation can become so important to parents that they allow it to dictate the hours they work and the time they take away from work. It has also been documented that some parents chose employment with hours that are suitable to work around their child’s sport schedules [209,210,214]. The following sections explore the effects of youth sport involvement on parents’ mental health.

#### 3.4.3. The Effects of Youth Sport on Parental Mental Health

Participation in youth sport can entail large commitments that impact the emotional well-being of parents, particularly if the child is involved in high-level athletics [194,203,209]. Research has documented that due to the investments and demands placed on parents of young athletes, these individuals have demonstrated high levels of stress and even burnout [4,195,215]. In a study by Dorsch *et al.* [4], parents spoke of negative emotional experiences they endured in relation to their child’s sport experience including, anger and frustration with their child/family, guilt (stemming from feelings of lack of control or from a lack of involvement in their children’s sport endeavours), and resentment of themselves and of the youth sport context. Parents also expressed feeling mentally and physically exhausted and as a result often experienced relief at any break in the sporting season [4]. While parents in this study also mentioned some positive experiences, this extends beyond the scope of this review.

##### 3.4.3.1. Parent Stresses in Multiple Athlete Families

It is challenging for parents and families to accommodate the needs of one child’s sport involvement; therefore, these demands are only increased in multiple child-athletes families. Indeed, it has been documented that parents with multiple children involved in sport experience further tension [12,198,216]. Research has indicated that having more than one child in sport or extra-curricular activities can prompt parents to split their focus and send parents in different directions with their children [209]. This reduces the amount of time couples and families spend together as a unit. Within these multiple child-athlete families, while sport was initially perceived as a disruption by the parents, it eventually became a way of life leaving parents feeling powerless and trapped by the demands associated with it [209,217]. Further, for some of these multiple athlete parents, the degree of support and sacrifice associated with their children’s sport involvement ended up exceeding their initial expectations prior to involvement in the youth sport environment [4].

Research has indicated that parents often feel tension with respect to the resources allocated to children within the family unit, whether athletes or non-athletes [12,191,192]. It is evident from the literature that siblings of sport-involved youth do not spend the same amount of time with their family, enjoy the same amount of attention, or have access to the same resources as their sport-involved sibling [12,203]. This is further outlined in the section on siblings below. Parents have also experienced stress related to the division of a family unit, seemingly forced to choose one child over another. In Fraser-Thomas *et al.*’s [12] chapter, it was indicated that parents experienced distress over the quality of their relationship with other children when one child is more involved in sport than others.

##### 3.4.3.2. Parents Taking on a Dual Role: Parent-Coach

As mentioned, parents’ roles in their child’s sport experience can range from providing transportation to and from events to being a coach or official [218]. While both parents typically play a role in the youth sport experience, the roles often differ between mothers and fathers. Research has shown that within youth sports, fathers are often expected to support and guide their children as they learn to play sports. Some fathers primarily provide financial support [12,219,220], while other fathers fulfill the guidance and advisory role by serving as teachers, coaches, managers, agents, mentors and advocate for their child athletes [187,194,210,221]. It has also been documented that fathers have utilized the sport context as an opportunity to spend quality time with their children [222]; however, there are some negative aspects associated with this involvement. It has been noted that fathers have struggled to separate the roles of being a parent and coach, particularly when dealing with their own child [187]. Further, some fathers have expressed feelings of judgment by their children for the amount of time they spend with them in sport contexts [187,223]. Volunteer coaching positions in sport programs are difficult to fill and often stressful, retained for only a short time [223,224,225]. McCann [226] indicated that parent-coaches can become worn out by parent complaints and their sometimes limited sports knowledge which impedes their ability to coach effectively. Moreover, this stress has led to decreased enjoyment in coaching and resulted in many parent-coaches dropping out of this volunteer position [227]. A study by Weiss and Fretwell [228] found that parent-coaches experienced many challenges including the ability to separate the parent-child relationship from the coach-player relationship, finding a balance between unfair behaviour, favouritism towards their own child and treating other players differently, and the uncertainty and ambiguity of their role as a coach. This is similar to findings in other literature on the subject, as McCann [226] found that while some parents enjoyed coaching their own child, they were uncertain about how they should treat their child in comparison to other children on the team. To avoid demonstrating favouritism, some parent-coaches would praise their child less and substitute their child more often than other team members, which in turn created strain on the parent-child relationship [226].

Related to a mother’s role as a coach in youth sport, there has been very little research conducted on this topic. Sport has been widely assumed to be a central context for fathering [220] and masculinity [229,230,231]; therefore, the cultural value placed on men’s sport and males as superior coaches and athletes negatively affects women’s opportunities as coaches and athletes [232]. Much of the research about mothers in sport tends to focus on women coaching at the collegiate level. In the sporting world, the lack of support systems, mentoring programs and networking opportunities may contribute to why the amount of women coaches is so low [221,229,231,232]; however, this topic is beyond the scope of this review.

##### 3.4.3.3. The Mother-Load

In today’s society, many mothers work full time outside of the home while also tending to many of the unpaid duties, including household tasks and childcare. While men continue to spend 53 minutes more per day engaged in paid work compared with women, women continue to take on the majority of unpaid tasks with an extra one hour and thirteen minutes more per day compared with men [233]. Particularly with respect to childcare, it has been documented that women spend more than twice as much time on this activity than men [233]. Therefore, when children participate in youth sport, these responsibilities add to a mother’s often full plate, even more so if the sport is at a competitive level.

Gender roles tend to be defining agents for the roles taken on by parents in youth sport [221]. This often entails mothers acting as “team moms” and fulfilling the roles of team director and chauffeur [194]. It has been identified that mothers’ roles include providing transportation, preparing meals, managing competition schedules, laundering uniforms, and serving as social directors for teams [12,194,214]. Furthermore, women continue to carry much of the responsibility that involves the planning, scheduling, and coordinating of their family’s activities [234]. Many mothers perceive themselves to be more supportive and involved in their child’s sport participation than their husbands, which adds to the mother-load [187]. When mothers try to meet expectations of mothering practices while also trying to meet their own individual needs, many women experience feelings of tension, conflict and guilt [235]. Some mothers sacrifice their career and social life to meet the needs of their children, particularly those who participate in sport [194,216]. The connection between children’s sport participation and being a good mother has led to the creation of an environment where a woman’s moral worth as a parent is evaluated by her ability to foster her children’s sport participation [194]. If these expectations are not met, mothers can feel as though they have failed their child(ren). Mothers, compared to fathers, often reduce their work hours or career goals to better accommodate their children’s lives, including their sport participation [12,194].

In a study by Wiersma and Fifer [35], parents indicated that in addition to the need to balance all of the roles related to youth sport practices (e.g., transportation, volunteer), they were also required to attend to other scheduled appointments, assist with homework, provide dinner, and ensure that their children went to bed at an appropriate time, which often caused additional stress on the parents. In an attempt to cope with these demands, parents within this study tended to limit their children’s sport involvement to one sport and restricted out-of-season activities [35].

#### 3.4.4. Effects on Parents’ Healthy Living Practices

With the amount of involvement that is required of parents in their child(ren)’s sport, it is not surprising that parents’ healthy living practices are affected. In a study by Grundtner and Koch [236], it was revealed that many parents tended to make their children’s extra-curricular activities the focal point of family life and alter their focus from activities for their own leisure and enjoyment to focus on activities of their children. This can be to the detriment of parents’ own healthy living practices, such as their eating habits and sport and leisure pursuits [210]. Moreover, Dixon [222] conducted a study that focused on working mothers which found that their child’s sport participation impeded their own physical activity participation. It was revealed that this was due to both the lack of perceived time and support they received. Dixon concluded that women struggle dividing time between work and family obligations including their children’s sport participation and finding time for their own physical activity pursuits [222]. Further, it has been found that eating dinner together as a family is often neglected due to the time demands or scheduling conflicts of youth sport [209,210,212,237]. For example, Trussell [212] found that during youth sport seasons, dinner was often eaten on the run. As mentioned earlier, a study by Chircop *et al.* [77] found that increased emphasis on participation in sports was associated with fewer meals at home and more family fast food consumption.

#### 3.4.5 Effects on Couplehood

Due to their involvement in their child(ren)’s sports, parents have been found to make sacrifices including forgoing personal and social time. This sacrifice has also included the time which parents spend as a couple [4,191,192,210]. It has been found that parents feel pressured by the wider culture to place their children’s needs over their own and those of their partnership [238], which can impact the marital dyad [203]. Despite this, parents of elite youth athletes continue to make the sacrifices they believe are necessary for their child to excel in the sporting world [4,203]. The negotiation of career-dominated marriages and juggling children’s participation in multiple extra-curricular activities can lead to considerable tension and marital strain [212]. A study conducted by Lally and Kerr [193] examined retirement from youth sport and the experiences parents had in sport. Parents indicated their children’s involvement in sport had often put serious strain on their marital relationship and only after the child’s retirement from the sport had parents began to re-establish these relationships.

Moreover, as noted above, some parents decide to take on coaching roles in youth sport, which adds another layer of investment (and thus less couple time); however, there is very limited research on this subject within the literature. Indeed few studies that touch on parental health and the commitment associated with youth sport discuss its impact on marriage relationships. Within these studies, it has been indicated that couples do lose time together [4,210]; however, few have explained the implications of this reduced time spent as a couple. Many parents believe that it is important to do whatever they can to help their child athlete pursue their athletic careers [203]. The following section outlines the effects of youth sport on siblings within the family unit.

### 3.5. The Effects of Organized Youth Sport on Siblings

Siblings are an important part of the family, and have been known for several decades to be key socializing agents for their other siblings [239,240,241]. The field of developmental psychology has provided a strong foundation in understanding relationships between siblings. Indeed from early research on this topic, it has been argued that the relationship between siblings is critical, particularly through the developmental years, as it tends to influence a number of different processes throughout youthhood. This relationship has been argued to have more influence than parental influence, as siblings tend to spend more time during childhood and leading into adulthood with siblings over parents [242,243].

While much is known on the general influence of siblings, prior to the last decade, research that investigated the influence of siblings on their athlete-brother/sister was sparse [242]. It has been found that siblings, whether younger or older, tend to have a large influence on physical activity and sport participation by acting as role models or rivals [8,242,244,245]. Early research examined the effects of birth order and gender and how these elements lead to sport participation and psychosocial outcomes [242]. More specifically, younger siblings tend to be the risk-takers that participate in sports that are considered to be more dangerous, while older siblings tend to avoid these environments [246,247]. Older siblings also tend to play more of a socializing role to their younger siblings in the sport context than vice-versa [248]. In Fraser-Thomas and colleagues’ [12] review of family influence on children’s sport involvement, the influence of siblings on youth athletes is discussed. The authors reviewed direct (e.g., child-sibling) and indirect (e.g., sibling-parent-child) influences that impact the overall experiences and perceptions of the youth athlete. However, there was no discussion on the inverse effects of how youth-athlete sport participations influence siblings within a family. As with parents, the effect of siblings in the youth sport context is reciprocal and the following section reviews psychosocial impacts of youth sport on siblings.

#### Psychosocial Effects

Côté [8] was one of the first researchers to explore the influence of youth sport on sibling relationships and dynamics. Findings from this research found both positive and negative sibling experiences, in which siblings were seen as motivators and role models for their brother/sister where cooperation emerged, but also in some cases caused bitterness and jealousy due to an uneven distribution of resources (e.g., time and money) within the family [8]. Findings from this study were reproduced by Kay [209] when she looked at elite child-athletes and the impact of sport participation on family life. This study indicated that some siblings were very supportive of their sibling’s sport involvement, while others conveyed negative emotions, including feelings of hurt and jealousy. From this study, it was also noted that parents seemed to be aware of the potential impact a child-athlete’s involvement may have on their siblings and expressed attempting to make an effort to ensure all children were treated on the same level [209]. In contrast, results from a qualitative study found that regardless of parents’ best efforts they were unable to treat all siblings equally in terms of attention and time, as parents tended to allocate more resources to the child-athlete in the family [200]. In Tressel’s [210] study, siblings were often noted as being “along for the ride”, indicating that parents placed value and investments on the talents of one child over another, which had potential detrimental effects on these children. Similarly, in another study, parents believed that bringing younger siblings who did not participate in sport along to practices and games of a sibling-athlete was a means of socializing with the other child participating in sport [35]. While some siblings enjoyed the experience and supported their sibling, others were less satisfied and found that the majority of parents’ focus was on the athletic sibling [35,209]. In line with this, Townsend and Murphy [237] highlighted that when younger siblings were brought to a sporting event, increased signs of irritability and fatigue were documented in these children. Lastly Newhouse-Bailey [203] yielded similar results to both Côté [8] and Kay’s [209] studies; however, what was unique about these findings were that siblings often felt that their autonomy was not being supported as their opinions and decisions were not taken into consideration, particularly in regards to attending practices and games of another sibling. Further, within this study, siblings not only scored low on affective involvement, indicating they perceived that all activities in the family were not equally valued, but also voiced concerns and the desire to spend more time together as a family, as the sport required the majority of time demands, limiting family time and causing stress to the overall family system [203].

It has also been documented that when a child in the family is thought to be the star athlete, the relationship between the child and his/her sibling(s) can tension [210]. Findings also found that these tensions between siblings further arose when the younger sibling was considered to be the elite athlete (versus the older one). Moreover, this tension also made siblings question their own abilities in the sport, particularly when skill level could be measured and compared between each other, and led to considerations of sport dropout [210]. Within this study it was also documented that siblings within a star-athlete family wanted to foster his/her independence by pursuing his/her own extra-curricular activity, linked with the creation of his/her own identity. In contrast, Côté [8] found that younger sibling or even twins displayed signs of bitterness and jealousy toward their older sibling’s sport achievement. It was outlined that when the family largely invested into one child’s sport it created “an uneven distribution of resources within the family, frequently resulting in feelings of tension from a sibling” [8]. These examples relate back to the notion that while a child can positively influence their siblings into sport participation, he/she can also negatively affect this element of a child’s life.

## 4. Discussion

### 4.1. Summary

**Figure 1:**
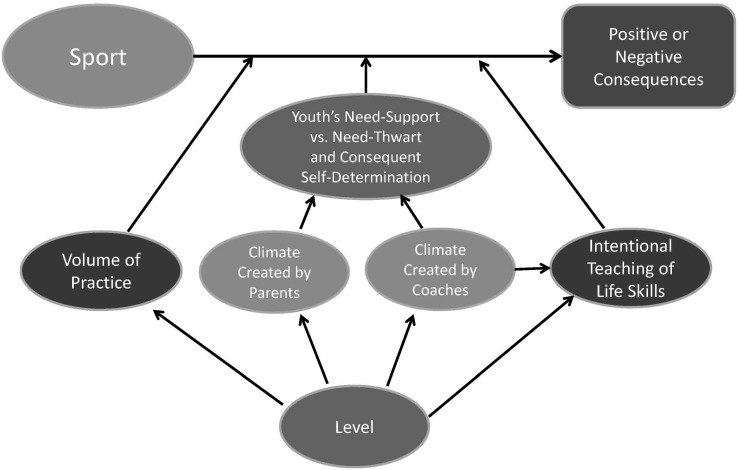
A proposed model to explain under which circumstances sport has a positive or a negative influence on youth.

This review examined current evidence on the negative physical and psychological effects of youth sport participation on the youth athletes, as well as impacts on their parents such as time and financial investments, parental stress and taking on multiple roles. Lastly, this review explored the negative psychosocial effects of organized sport on siblings within the family unit. With respect to the youth athlete, it was revealed that some sport contexts bring about negative outcomes, such as injuries, concussions, and dangerous weight control practices. With regards to youth mental health, increased alcohol consumption has been linked to sports practice (particularly in certain team sports), as well as decreases in well-being with high levels of training, which likely leads to burnout and dropout. Côté *et al.*’s [8,249] DMSP proposed that not all organized sport experiences are created equal, and several reviews have shown that early specialization carries the most risks in terms of negative physical and mental health consequences [18,26,46]. This is likely related to the high level of practice volume. Moreover, this review has shown that it is not only the amount of sport practice, but the motivational climate created by coaches and parents that impact youth outcomes in sport and thus these factors are likely important moderating influences between sport participation and youth athlete consequences. From this review, the authors propose a model to account for the outcomes of organized sport (see Figure 1).

This model builds from Côté’s Development Framework for Sport which proposes that the context is an important determinant as to whether youth athlete development is optimized or not (see [250]). It is also inspired by SDT and AGT which emphasize the importance of the motivational climate. Recently Duda [251] has proposed a new taxonomy that integrates SDT and AGT and contrasts two motivational climates, one that is empowering defined as being autonomy supportive and task-oriented and one that is disempowering, that is controlling and ego-oriented. Within this model (Figure 1) and stimulated by this review, three important contextual factors are proposed that will moderate if there is a positive or negative consequence from sport participation: (1) the volume of training, (2) the climate (empowering *vs.* disempowering), and (3) if there is intentional teaching of life skills or not. All three variables are influenced by the level of sport (e.g., the pathways—recreational, competitive, elite). The early specialization pathway/elite level will lead to high volumes of training, more disempowering motivational climates and less intentional teaching of life skills which would all contribute to sport having more of a negative effect on youth physical and mental health. On the other hand, recreational participation or less competitive sampling would lead to more moderate volumes of training, more empowering motivational climates and purposeful teaching of life skills which would likely result in positive youth development outcomes. We propose also that coaches that are more autonomy supportive and task-oriented (empowering) are also likely those that value life skills and intentionally teach them.

Within the following sections limitations and current gaps in the literature based on this review are discussed. From this, calls for future research and practical implications are discussed.

### 4.2. Limitations

Some of the limitations in the current review should be noted. While a comprehensive search was conducted and 186 articles were reviewed, this literature review does not represent all of the research on the impact of organized sport on the family, as it is impossible to obtain all literature on the topic, particularly related to the negative physical and psychological effects of sport on the youth athlete, as there are thousands of studies on this topic. As such, a review of literature within the past two decades was conducted to allow for a manageable review. Specifically, the most recent and pertinent articles were secured related to specifically the negative physical and psychological effects on the youth athlete. Further, as mentioned, due to the length and complexity of this paper, social impacts of organized youth sport were not comprehensively reviewed within this paper, despite the inevitability of this topic within the sibling section. Additionally, this review does not discuss research surrounding the influence and/or impact of teammates on the youth athlete. While very important, this exceeds the scope of the review. Further, author interpretation and judgment were involved in evaluating the quality of different studies. While co-authors discussed and agreed upon studies, there is room for interpretation, which may lead to other interpretations of the data. Moreover, as there is a dearth of literature surrounding the physical health of parents related to their child’s sport involvement, this section was lacking within the review. Within the current literature, there is also a lack of empirical literature on the on how organized sport affects the inter-personal dimension of the family unit and therefore was lacking within this study. Moreover, as many of the studies revealed from our comprehensive literature search were from a North American context, this review illustrates many pertinent issues that are relevant to the family unit within Canada and the United States and may not necessarily be generalizable to family units within sporting contexts in other parts of the world. Lastly, many of the reviewed studies are cross-sectional and some were qualitative in-nature, therefore making it difficult to infer causality between sport and the identified outcomes.

### 4.3. Areas for Future Research

First and foremost, research should be conducted on the proposed model within this review to verify if indeed the three proposed contextual factors moderate the link between sport participation and positive and/or negative youth outcomes within this environment, and perhaps examine which, if any, are most influential. Also, research should aim to understand if early specialization leads to decreased sport self-determination and subsequent negative outcomes; although this has been postulated, it has yet to be proven. Indeed a study comparing different levels of youth ice hockey (from recreational to elite) within Canada on mental health and determining if self-determination mediates the relationship is underway [252].

The focus of this review was on the individual actors within the family unit, as the inter-personal dimension of understanding the effects of youth sport involvement on the family unit as a whole is lacking. Therefore, future research is required in this area. While the youth athlete has been heavily studied, parents and siblings have been somewhat neglected in the research when exploring the effects of youth sport participation [12,215]. It would be critical to look specifically at the individual actors that make up the family dynamic (athlete, parents, siblings) and understand the impact of the youth sport context on actors’ physical and mental health individually and together as a family unit. More specifically, parents are also influenced by their child’s sport participation (e.g., [4,192]); therefore, understanding both individual (e.g., age, temperament) and contextual (e.g., community involvement, individual *vs.* team sport) factors that affect this socialization process would provide insight into such interactions [12,214]. Further, Dorsch [215] identified that there is a limited understanding of how parents make sense about the role of youth sports in shaping not only family relationships, but also parenting practices; therefore, future research is warranted in understanding parent’s perceptions of the role of youth sport in the family context. Moreover, the impact on family interactions, dynamics, and relationships suggests the importance for future research in this area*.* Côté [8] argued that “without doubt, the family conditions that emerge…are an incomplete picture of the family dynamics of talented performers”, which explicitly highlights the need for future research in this area. Specifically, researchers should conduct longitudinal studies that follow sport-based families during multiple time points throughout a season (e.g., pre-season, in-season, post-season) to compare well-being of actors within a family at these different stages. Fraser-Thomas and Côté [253] discussed the importance of longitudinal research in this field that investigates the long-term impact of youth sport involvement on the family environment, specifically looking at interactions between these youth athletes and their families over time. For example, a multiple case study approach studying different families at different levels of sport and different sport contexts could be highly interesting when understanding its connection to family life in order to attain a more comprehensive understanding. As Wolfenden and Holt [219] argued, it is important to explore and compare these family influences across different ability levels within the sport environment and over time, as the child moves through adolescence.

As parents are becoming increasingly busy, taking on many roles within their lives, it would be worthy to look at parents’ in a triple role as a parent, work professional, and youth sport coach, which is important to not only understand the effects this would have on family dynamics, but also to shed light on the well-being of the adult involved in these multiple roles. More specifically, understanding elements of stress and burnout, life-balance, and well-being of parents involved in youth sport would prove to be fruitful, as well as understanding the stress youth sport places on the marital-dyad. For example, a qualitative study exploring impact of organized sport on parents’ well-being, healthy living practices and couplehood would be novel and comparing parents of different levels (recreational, competitive and/or elite) would be a particularly effective approach. Also, it would be critical to delve into the role of mothers in particular, as limited research has investigated women in the roles of mothers of youth athletes and sport coaches.

Within Trussell’s [210] study, she discussed the ‘loss of family togetherness’ due to youth sport participation; therefore, future research needs to further understand the effects of youth sport involvement on family relationships, including time spent together. Using daily diaries from multiple family members could be a fruitful method to investigate this. From this review, there is a lack of literature surrounding the effects on parental physical health. For example, it is evident that literature surrounding healthy eating habits of families involved in youth sport is noticeably absent. Future research is warranted that assesses family nutrition practices and perhaps their physical activity behaviours using quantitative measures, such as logs. Further, it is important to document these behaviours longitudinally to compare in and out of season habits. Similarly, the effect of sport on both youth athletes’ and parents’ sleeping habits needs to be investigated (e.g., how sleep relates to physical and mental health at different levels of sport involvement), as sleep has been found to play a key role in health promotion and has been found to be compromised in high level sport with athletes not having enough time balancing life demands. Screen time is another variable that would be important to investigate. For instance one could study if it is healthier for an athlete to do one hour of highly competitive sport and then spend the rest of his/her day on screens or is it better to have youth sample a variety of recreational and lifestyle physical activities in the day and have less screen time.

While initial progress has been made in uncovering the influence of siblings in the sport environment, there is much work to be done. There has been a dearth of research looking at the reciprocal influences of siblings and youth athletes. It is critical to investigate the impact of youth sport participation on siblings, both those who are and are not involved in sport themselves, and understand siblings’ well-being and position within the family unit. As noted by Partridge *et al.* [242], researchers should aim to understand how sibling relationships are affected when one individual is involved in sport at a highly competitive level. Tressel [210] and Newhouse-Bailey [203] are two of the few studies that have qualitatively examined the influence of sport participation on the family, including both the positive and negative consequences it has on siblings. However, to the authors’ knowledge, there has never been a study conducted that solely examined the effects of youth sport participation on siblings. Therefore, it would be important to understand how such involvement affects siblings’ motivation, mental health, identity, and how this might be moderated by parents’ behaviours.

Previous research that has touched on the impact of youth sport engagement on the family unit has predominantly been qualitative. While using qualitative methodologies are very important to understand in-depth feelings and experiences, quantitative methods or mixed-methods designs should also be utilized to accurately measure effects of youth sport on the individual actors in the family unit. Finally, research conducted in this area has typically focused on families in which there was typically one elite athlete who stood out above the other siblings; therefore, research should look at families that have multiple siblings playing sport at elite levels and how this impacts the psychosocial development of each individual and the family as a whole.

### 4.4. Practical Applications

A number of concerns surrounding the effects of sport on the family unit were discussed within this review. From these, practical applications are inferred in this section. First, recommendations are made surrounding the physical effects of youth sport; and second, the importance of optimizing the sport experience for all actors within the family unit is emphasized. At the broadest level, and stemming from the proposed model, it is recommended that in order to ensure the most positive outcomes for youth: (1) moderate volumes of training (practices and competitions) need to be encouraged; (2) empowering (autonomy supportive and task-oriented) climates need to be fostered by coaches and parents; and (3) intentional teaching of life skills need to be incorporated with physical training. These are discussed in further detail below.

#### 4.4.1. Physical and Safety Recommendations for Youth Athletes

As stated by Merkel [34], “changing the future of youth sports for the better needs a collaborative effort between parents, coaches, teachers, health professionals, community leaders, and politicians”. As parents play a critical role in the sport socialization process of their children [190], they take on an imperative role in this process. Before parents enroll their child(ren) in sport, it is vital that they become informed of both the benefits and dangers associated with various developmental pathways of sport participation. For example, it has been recommended that educational opportunities are created for athletes, parents, and coaches to provide information about youth safety related to specialization and overtraining, but also regarding proper nutrition and fluids, including the dangers of extreme dieting, and sleep patterns [46,95,98].

While many educational tools and recommendations have been developed, such as creating “Return to Play” guidelines [254], more preventative steps need to be taken to ensure these policies are followed to protect the well-being of youth athletes, such as creating rules and legislation and reinforcing that organizations’ mission and values are carried out. Initial steps have been taken to physically protect youth athletes, particularly through the implementation of a preparticipation physical evaluation (PPE) that aims to aid in the reduction of injuries, provide athlete education, and identify serious health problems, which may be beneficial in keeping athletes safer while participating in sports [255]. Merkel [34] also suggested that an assessment of readiness should be performed to assist in determining if a child is prepared to enroll in sport and at which level of competition the child can successfully participate. It has also been suggested that active prevention measures be put into place for therapy and training programs of young athletes, thereby decreasing the rates of injury and re-injury [256]. While these are important first steps, much work is still needed. For example, the use of the PPE and the assessment of readiness is absent within Canadian sport organizations and would be beneficial in assessing the physical, emotional, and psychological readiness of youth involved in the sport environment. It has also been recommended that national governing bodies for youth sport and youth sport organizations assume evaluations investigating the effectiveness of age-appro­priate techniques, rules, and demands (including playing and practice standards) in reducing sports-related injuries [3,18,53,64].

From the above literature, it is also evident that weight control in sport is an ongoing issue for some youth athletes and that all actors involved should be educated and prevention programs be developed, implemented and evaluated [182,257]. Specifically, parents, coaches, and athletes who are involved in weight-regulated, endurance or aesthetic sports should be educated on safe and healthy methods of dealing with the weigh-ins and other pressures that can arise in these environments. Given that eating disorders are most prevalent during adolescence [103], occur predominantly in weight cutting sports (e.g., wrestling, gymnastics, boxing), and are more common among athletes than non-athletes [84,99,100,101], special care (e.g., participation in education program) needs to be taken when dealing with individuals who fall into these demographics.

As the competitive youth sport environment often requires many hours of involvement each week, a re-evaluation of the sport environment may be warranted at a policy level to consider fewer hours for practice as it places a strain on the young athletes, their parents and families alike. In line with this, Luke *et al.* [48] concluded that when scheduling youth sporting events, activity volume and intensity should be considered over a 48-hour period to optimize safety including the recovery time between training and competition bouts and the time between competition and sleep (≥7 h). Brenner [46] recommended that youth athletes should have one or two days off per week from their competitive or sport-specific training to allow for adequate physical and psychological recovery. Additionally, Jayanthi *et al.* [18] recommended that youth athletes should not spend more than twice as much time playing organized sports as they spend in other physical activity pursuits, such as physical education class and unorganized play with friends. As previously mentioned, Merglen *et al.* [124] found that well-being decreased in youth athletes when participating in more than 14 hours of sport per week; therefore, policy makers, sport organizations, parents, and coaches should ensure that sporting schedules (practices and competitions) of youth athletes do not exceed 14 hours per week. On a larger scale, Brenner [46] discussed that youth should have at least two or three months off from a specific sport each year for the same reasons. Lastly, all youth athletes should be encouraged to participate in a few recreational sports at an early age for diversification while keeping in mind the recommendation to not exceed 14 hours of sport per week.

#### 4.4.2. Optimizing the Sport Experience for the Family Unit

Youth sport should be an environment that emphasizes fun, while maximizing physical and psychosocial benefits of participants [34,120,121]. While several youth sport organizations promote these values within mission statements, “win-at-all-costs attitudes and overwhelming emphasis on outcomes…has permeated youth sport” [258]. This overwhelming emphasis on achievement regardless of the consequences has become such an issue within Canada that an online course has been created, “Respect in Sport”, that encourages adults involved in youth sport to consider their behaviour through the completion of a one-hour online parent program focused on helping parents set reasonable expectations for their children in sport [259]. While the development of such programs indicates progressive steps in the area of parent education, little research has been conducted to evaluate the effectiveness and impact of such programs on the youth sport environment. Further, it is recommended that an education program, such as ‘Respect in Sport’, become a mandatory prerequisite for all parents and coaches involved in youth sport across North America prior to the commencement of a youth sport season. These education programs should make specific recommendations to foster an empowering and task-oriented climate. Moreover, given that the goal of youth sport programs should be to encourage individuals to be healthy and active across the lifespan, and that the emphasis should be on fun, skill acquisition, sportsmanship, and safety, it appears as though early specialization pathways are often falling short of these important objectives [48].

With regards to coaching specifically, Camiré *et al.* [260] provided five recommendations for facilitating positive youth development outcomes that include: (1) carefully developing a coaching philosophy, (2) developing meaningful relationships with athletes, (3) intentionally planning developmental strategies in coaching practice, (4) talking about and having athlete’s practice life skills, and (5) teaching athletes how to transfer life skills to non-sport settings. Similarly, Côté, Strachan, and Fraser-Thomas [261] provided six recommendations made for youth development based on the DMSP that entails: (1) sport participation should include interactions between youth and between youth-adults; sampling and play should take place (proximal processes), (2) youth should have an opportunity to choose to specialize or continue to sample based on proximal processes, (3) developing assets of the youth athlete should be a priority to coaches and parents involved in sport experience, (4) the eight setting features of National Research Council and Institute of Medicine should be implemented to provide a context that encourages life skills development which include (a) safe and health-promoting facilities, (b) clear, consistent rules and expectations, (c) warm, supportive relationships, (d) opportunities for meaningful inclusion and belonging, (e) positive social norms, (f) support for efficacy and autonomy, (g) opportunities for skill building, and (h) coordination among family, school, and community (see National Research Council and Institute of Medicine [262] for complete description of setting features), (5) youth sport programs must be designed and implemented with consideration for healthy youth development over time, and (6) coaches and parents play critical roles in youth sport experience; therefore, appropriate training that includes positive youth development principles should be provided to all adults leaders and coaches.

Within Canada, at both the national and provincial levels, sport organizations (e.g., Own the Podium) are funded based on early talent identification searching for talent at younger ages and to fewer athletes; therefore, the developmental model of broad-base participation has been neglected [263]. In many regions across North America, funding to municipal, school-based, and after-school sport programs are available solely to the youth athletes whose parents can pay for their registration, equipment, travel, and coaching [263]. Therefore, focusing more on the large-scale, barrier-free participation within youth sport with a focus on enjoyment and the development of physical and life skills should be prioritized. If such opportunities were put in place, more youth of all socio-economic levels would have access to positive sport experiences, enabling more youth to live healthier and more productive lives.

In line with Harwood and Knight [213], it can be proposed that parents select appropriate sporting opportunities, such as less competitive sport for younger children and provide necessary types of support. For example, providing positive emotional support and constructive informational support, such as praise may be effective [264]. Through understanding and applying appropriate parenting styles, parents can avoid placing unnecessary stress and/or pressure resulting in negative sport experiences for youth [264,265]. As research has identified both large financial [35,196] and time commitments [35,187,196] due to the demands of youth sport, parents should be informed that youth sport involvement can be both physically and emotionally taxing for themselves, as well as stressful for the family unit as a whole. In a position paper, Harwood and Knight [213] recommended that a parent’s ability to cope with various life demands by means of a variety of intrapersonal, interpersonal and organizational skills and strategies can be noted as a characteristic of parenting expertise (see [213] for full review). Specifically, “sport parenting expertise requires the development and application of various coping skills and strategies to manage these diverse demands” [213]. Ultimately, parents would benefit from considering that if their child’s sport participation is a source of stress rather than enjoyment, a re-evaluation of where human and financial resources are distributed within the family, may be better for the well-being of the family unit in the long run. From this, delivering workshops that provide strategies and recommendations surrounding this topic would be of benefit for parents of youth athletes. Therefore, it is recommended that parents should be honest with themselves in terms their child’s sport abilities and the financial investment it requires [264] and try to seek balance in their life, which includes ensuring there is quality time with all of their children and their spouse [266]. Lastly, parents should consider the well-being of all of their children within the family unit and attempt to distribute resources (*i.e.*, time, financial, human) equally between children regardless of their involvement and level of sport participation.

## 5. Conclusions

In many families, the culture of sport has become a defining characteristic, giving the family unit a sense of identity [203]. While it is evident that organized sport serves as a tool to bring the family together and promotes positive outcomes in youth, it can also have detrimental effects on youth athletes, parents, and siblings. The purpose of this review was to shed light on the potential negative effects organized youth sport has on the individual actors within the family unit. From this review, it is evident that there are concerns that need to be further studied, such as worrisome physical and psychological effects on the youth athlete, the straining financial and practical investments that parents undertake with having child(ren) in organized sport, often leading to issues surrounding parental mental health, and psychosocial effects on siblings such as feelings of resentment and jealousy towards their brother or sister. Before parents enroll their child in sport, it is vital that they become informed of not only the benefits, but also the dangers associated with sport participation. With much literature indicating an overall increase in stress in youth and adults, this review raises concerns surrounding the issue of organized youth sport and whether involvement helps youth and families flourish or simply causes stress to the family unit. Further, a framework has been proposed (see Figure 1) that could potentially fuel future research and direct interventions to optimize the youth sport experience. In conclusion, the negative effect youth organized sport participation has on the individual actors of a family has not yet been fully explored or defined; therefore, future research should utilize this review as a framework in which to build from. The darker side of sport needs to be further studied and addressed so that not only sport participants can thrive, but so too can their families.
